# Traditional Chinese medicine for heart failure with preserved ejection fraction: clinical evidence and potential mechanisms

**DOI:** 10.3389/fphar.2023.1154167

**Published:** 2023-05-10

**Authors:** Yujian Fan, Zhihua Yang, Lin Wang, Yangxi Liu, Yulong Song, Yu Liu, Xianliang Wang, Zhiqiang Zhao, Jingyuan Mao

**Affiliations:** ^1^ National Clinical Research Center for Chinese Medicine Acupuncture and Moxibustion, First Teaching Hospital of Tianjin University of Traditional Chinese Medicine, Tianjin, China; ^2^ Institute of Traditional Chinese Medicine, Tianjin University of Traditional Chinese Medicine, Tianjin, China

**Keywords:** heart failure with preserved ejection fraction, traditional Chinese medicine, clinical evidence, potential mechanisms, cardiovascular disease

## Abstract

Heart failure with preserved ejection fraction accounts for a large proportion of heart failure, and it is closely related to a high hospitalization rate and high mortality rate of cardiovascular disease. Although the methods and means of modern medical treatment of HFpEF are becoming increasingly abundant, they still cannot fully meet the clinical needs of HFpEF patients. Traditional Chinese medicine is an important complementary strategy for the treatment of diseases in modern medicine, and it has been widely used in clinical research on HFpEF in recent years. This article reviews the current situation of HFpEF management, the evolution of guidelines, the clinical evidence and the mechanism of TCM in the treatment of HFpEF. The purpose of this study is to explore the application of TCM for HFpEF, to further improve the clinical symptoms and prognosis of patients and to provide a reference for the diagnosis and treatment of the disease.

## 1 Introduction

Heart failure (HF) with preserved ejection fraction (HFpEF) refers specifically to left ventricular ejection fraction (LVEF) ≥ 50%. It is a syndrome that accumulates in multiple organs and systems. The main clinical manifestations are exertional dyspnoea and exercise intolerance. According to the latest American Heart Association/American College of Cardiology/Heart Failure Society of America (AHA/ACC/HFSA) guidelines in 2022, heart failure is divided into four categories: heart failure with preserved ejection fraction (HFpEF), heart failure with reduced ejection fraction (HFrEF), heart failure with midrange ejection fraction (HFmrEF) and heart failure with improved ejection fraction (HFimpHF) (Heidenreich et al., 2022). According to the epidemiology in 2019, cardiovascular disease (CVD) accounted for approximately 45% of the causes of death in China, and 2 out of every 5 deaths were from CVD. At present, the number of CVD cases is 330 million, of which heart failure accounts for 8.9 million (Wang, 2021), while HFpEF accounts for between 19% and 55% of HF cases (Teramoto et al., 2022). The risk of death and rehospitalization from HFpEF is similar to that of HFrEF, and its prevalence rate is increasing by 1% per year (Paulus and Tschöpe, 2013). The distribution of HFpEF in the population is more in the United States than in Europe and more in Europe than in Asia. It is more prevalent in women than in men and in the elderly than in the young. With increasing age, the number of comorbidities in patients with HFpEF also increases (Dunlay et al., 2017). Therefore, in hospitalized cases of HFpEF, we cannot only consider cardiovascular problems, which results in the complexity and diversity of doctors’ diagnoses and treatment. The pathogenesis of HFpEF results from impaired cardiac filling and changes in the diastolic function of the left ventricle. In the past, primary myocardial injury was considered to be an important factor triggering HFpEF. Recent studies have shown that myocardial injury in HFpEF is not primary but rather systemic inflammation caused by arterial hypertension or metabolic diseases such as diabetes and obesity. It eventually leads to coronary artery microvascular inflammation, dysfunction, subendocardial ischaemia, myocardial cellular mechanisms and metabolic changes (Heinzel and Shah, 2022).

The 2022 AHA/ACC/HFSA guidelines proposed guideline-directed management and therapy (GDMT) as the cornerstone of drug treatment, and SGLT2i, MRA, and ARNI are included in the recommended drugs according to the guidelines. However, a one-size-fits-all treatment strategy has difficulty solving the real problem. For example, the risk period for HFrEF requires initiating drugs to prevent disease progression, while there is a lack of treatment strategies for the risk period for HFpEF. Given the high mortality and rehospitalization rate of HFpEF (Schrutka et al., 2022), how to use drugs and improve the prognosis of patients has also become the focus of our attention. The current treatments in Western medicine include SGLT-2 inhibitors, which can regulate diabetic comorbid disease and improve the quality of life of patients (Heinzel and Shah, 2022); sacubatril valsartan, which can prevent the deterioration of renal function and reduce the risk of cardiovascular death (Cui et al., 2022); corresponding anti-inflammatory strategies, which can improve systemic microvascular inflammation (Omote et al., 2022); pirfenidone, which can reduce TGF-β and improve myocardial fibrosis (Lewis et al., 2021); and spironolactone, which can regulate cardiac function (Beldhuis et al., 2021). Although its treatment takes into account the comorbidities and prognosis of HFpEF, it also has some limitations. For example, the clinical effect of some drugs is not obvious, or the regulatory mode is relatively simple. Clinical studies have shown that sildenafil (Lam et al., 2018) has no significant effect on haemodynamics and exercise ability, the effect of eplerenone on follow-up mortality is not clear, and the efficacy nitrate of quality of life cannot be evaluated due to the lack of long-term trials (Dong, 2022). Therefore, more effective measures are still needed to intervene in the development of the disease (Zhang et al., 2022). Previous studies have shown that traditional Chinese medicine can reduce the clinical symptoms of HFpEF, improve the quality of life of patients, enhance overall motor function and prevent the development of HFpEF (Bai, 2022; Tan, 2022). This study summarizes the management process of HFpEF and the clinical evidence and mechanism of traditional Chinese medicine in the treatment of HFpEF and reveals the clinical significance and development prospects of TCM in the treatment of HFpEF.

## 2 Management strategies for HFpEF

In the past, HFpEF was regarded as an early stage of the transition to HFrEF. Modern research shows that the transition from HFpEF to HFrEF is rare. Compared with HFrEF, HFpEF has higher comorbidity and noncardiovascular mortality (Heinzel and Shah, 2022). Therefore, the guide proposes different management models for HFpEF and HFrEF.

At present, HFpEF lacks a specific diagnostic test. Although Doppler echocardiography can support the diagnosis of HF through evidence such as increased ventricular filling pressure or decreased cardiac output, it cannot support the diagnosis of HFpEF (it cannot reflect LVEF or cardiac morphology) (Dunlay et al., 2017). When physical examination, imaging data, and blood tests are unable to provide a clear basis for diagnosis, an invasive exercise test is necessary (Borlaug, 2020). This is undoubtedly a difficult problem in the process of HFpEF research. From the point of view of treatment, the treatment strategy of HFrEF cannot be fully applied to HFpEF, mainly because there is no symptomatic treatment for the aetiology of HFpEF. Some HFpEF patients complain of chest discomfort, which suggests that the disease may be related to coronary artery disease (Rush et al., 2021), abnormal cardiac metabolism (Mishra and Kass, 2021), and myocardial injury. Dizziness and orthostatic intolerance suggest that HFpEF may be caused by cardiac amyloidosis. Peripheral oedema, abdominal distension, loss of appetite, and early satiety are usually symptoms of late HFpEF complicated with right heart failure. In addition to the aforementioned intractable symptoms, complications closely related to HFpEF should also be considered. A history of atrial fibrillation (Reddy et al., 2018), obesity, diabetes, or metabolic syndrome significantly increases the likelihood of HFpEF. Studies have shown that strategies to relieve cardiac congestion can reduce the hospitalization rate of HFpEF (Borlaug, 2020). Strategies include the use of glucocorticoid receptor antagonists, the management of complications, and the promotion of a healthy and active lifestyle. HFpEF is an important issue in the cardiovascular field. In the future, HFpEF will be more strictly classified to achieve individualized treatment.

## 3 Evolution of guidelines

**FIGURE 1:**
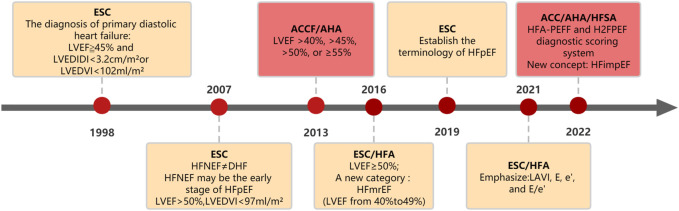
Evolution of guidelines for HFpEF.

As shown in Figure 1, the European Diastolic Heart Failure Research Group proposed in 1998 that the diagnosis of primary diastolic heart failure needs to meet three necessary conditions at the same time: 1) signs or symptoms of pulmonary congestion, 2) evidence of left ventricular abnormality and 3) left ventricular systolic function being normal or only mildly abnormal, with a left ventricular ejection fraction ≥45% (Group, 1998). In 2007, the European Society of Cardiology (ESC) proposed that heart failure with normal ejection fraction (HFNEF) cannot be equated with diastolic heart failure (DHF), and systolic heart failure (SHF) can also have diastolic dysfunction. The consensus is that HFNEF may be the early stage of HFpEF, and the following should be satisfied: 1) signs or symptoms of congestive heart failure, 2) evidence of left ventricular diastolic dysfunction and 3) normal or mildly abnormal left ventricular systolic function (Paulus et al., 2007). Since then, HFpEF and HFNEF have been widely used as terms in North America and Europe, and some people are opposed to using HFpEF instead of HFNEF (Sanderson, 2014). In the 2013 American College of Cardiology Foundation/AHA (ACCF/AHA) guidelines (Yancy et al., 2013), standards are proposed for the definition of HFpEF syndrome, including 1) clinical signs or symptoms of heart failure, 2) evidence that left ventricular ejection fraction is preserved or normal and 3) evidence of abnormal left ventricular diastolic function that can be determined by Doppler echocardiography or cardiac catheterization. HFpEF is classified as EF > 40%, >45%, >50%, and ≥55%. The 2016 ESC/Heart failure Association (HFA) guidelines define HFpEF as ≥ 50% (Ponikowski et al., 2016). HF includes a new category called HFmrEF, with an ejection fraction in the range of 40%–49%. In 2019, the ESC formally established the terminology of HFpEF, and its diagnostic conditions have also been updated: diagnosis based on the HFA-PEFF scoring system, including initial evaluation, UCG, and natriuretic peptide scores, functional tests, and aetiological diagnosis (Pieske et al., 2019). In 2021, to promote the wide application of the guidelines, the previous comprehensive diagnosis was simplified, emphasizing some clinically available variables: LA volume index (LAVI) > 32 mL/m^2^, mitral E velocity >90 cm/s, septal e’ velocity <9 cm/s, and E/e’ ratio >9 (Mcdonagh et al., 2021). In 2022, the ACC/AHA/HFSA announced two scoring systems. In addition to the previous HFA-PEFF diagnostic scoring system, the H2FPEF scoring system identified risk factors for HFpEF: hypertension, obesity, atrial fibrillation, pulmonary hypertension, advanced age, and average filling pressure height. The guidelines also proposed a new classification of HFimpEF. It is unclear which HFimpEF patients will be classified as HFpEF or HFmrEF, and the evidence for the treatment of HFimpEF is limited (Heidenreich et al., 2022).

## 4 TCM for HFpEF treatment

### 4.1 Understanding HFpEF in TCM theory

Originally, in TCM theory there was no separate classification of HFpEF or even HF. The disease is treated solely based on symptoms. During the Western Jin dynasty, the concept of HF was mentioned for the first time in the “Mai Jing” written by Wang Shuhe (Wang, 2019): “If heart failure falls, the pulse sinks.” In modern times, the TCM syndrome types of HF can be divided into *qi* deficiency and blood stasis, *qi* and *yin* deficiency, *yang qi* deficiency and blood stasis, and exhaustion of *yin* and *yang*. At present, there is no exact TCM syndrome for HFpEF, and its basic pathogenesis is deficiency in origin and excess in superficiality. In terms of aetiology, different doctors hold different opinions. Liu Jing (Liu and Xu, 2017) believes that the main cause of HFpEF is *qi* deficiency and blood stasis accompanied by *qi* stagnation and blood heat. Zhao Zhiqiang (Zhao et al., 2018) believes that the origin is mainly *yin* deficiency often combined with *qi* deficiency and yang deficiency, and the superficiality is mainly blood stasis, phlegm, and heat accumulation. Ji Hongyun (Ji et al., 2020) believes that although there are many causes of HFpEF, *qi* deficiency and blood stasis always underlie the disease. Shen Bin (Shen, 2018) believes that the basic pathogenesis of HF is the deficiency of heart *qi* and heart *yang*, while blood stasis is the key link, and other pathological factors are phlegm and water.

### 4.2 Clinical evidence of TCM for HFpEF

**FIGURE 2:**
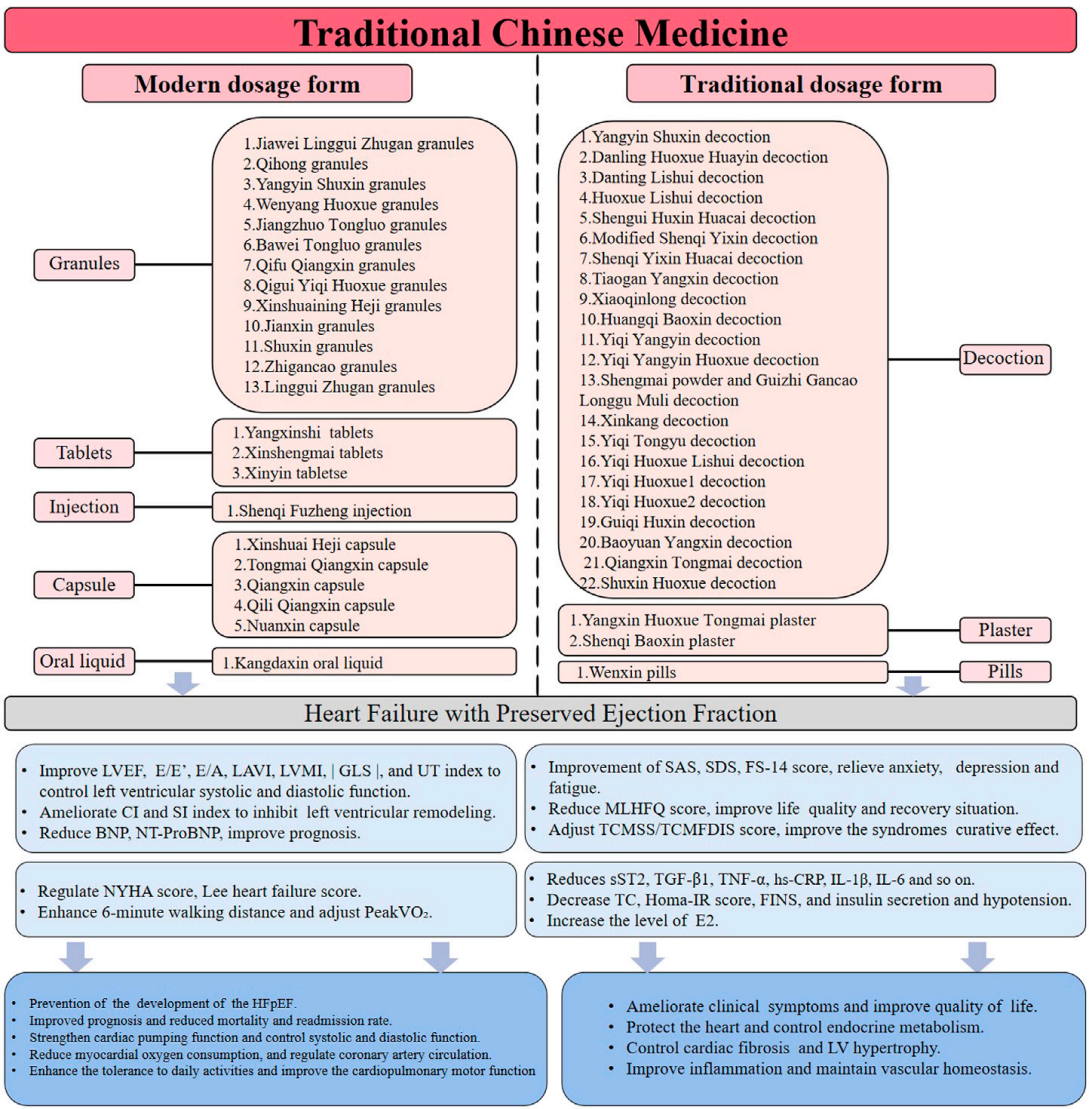
Clinical evidence of TCM for HFpEF.

In this paper, 58 clinical randomized controlled trials were collected by VIP, Wanfang, CNKI, SinoMed, PubMed, Embase, Web of Science, Cochrane, and clinical trials. At present, there are a large number of experiments on traditional and modern Chinese medicine dosage forms, as shown in Figure 2. Traditional Chinese medicine dosage forms include decoctions, pills, powders, and plasters. The dosage forms of modern traditional Chinese medicine also include granules, capsules, tablets, suppositories, injections, and so on (Nie et al., 2021). In this paper, 22 different TCM decoctions, 2 different plasters, 1 different pill (Tan, 2022), 13 different granules, 5 different capsules, 3 different tablets (Huang, 2005; Zhang, 2011; Huang et al., 2014; Liu, 2020; Zhu, 2021), 1 different injection (Xu et al., 2020), and 1 different oral liquid (Lin et al., 2016) are discussed as clinical evidence. Supplementary Table S1 analyses and summarizes the clinical evidence of TCM in the treatment of HFpEF, including 1) TCM dosage form, 2) clinical manifestations, and 3) clinical indicators. Based on Supplementary Table S1, Table 1 discusses the experimental mechanism of TCM in the treatment of HFpEF.

**TABLE 1:** Experimental mechanism of TCM in the treatment of HFpEF.

No	TCM	Composition	Proportion (people)	Quality conrtol report	Animal	Model	Dosage (kg·d)	Indicator	Mechanism	References
1	Yiqi Xiefei decoction	*Astragalus mongholicus Bunge [Fabaceae; astragali radix praeparata cum mell], Morus alba L.[Moraceae; cortex mori], Descurainia sophia L.) Webb ex Prantl [Brassicaceae; descurainiae semen, lepidii semen], Lycopus lucidus* var. *hirtus (Regel) Makino and Nemoto [Lamiaceae; lycopi herba], Wolfiporia cocos (Schw.) Ryv. and Cilbn. [Polyporaceae], Pueraria montana* var. *lobata (Willd.) Maesen and S.M.Almeida ex Sanjappa and Predeep [Fabaceae; puerariae lobatae radix]*	15:12:15:15:30:15	Prepared by Dongzhimen Hospital, Beijing University of Chinese Medicine (Kang Ren Tang).	SD rats	Abdominal aorta coarctation method	10 mL	2.LW↓.3.HR↓.6.Fibrosis of the left ventricle↓, Average fibrotic area↓.	②③⑥	Wang (2019)
2	Yiqi Huoxue granules	*Carthamus tinctorius L. [Asteraceae; carthami flos], Salvia miltiorrhiza Bunge [Lamiaceae; salviae miltiorrhizae radix et rhizoma], Astragalus mongholicus Bunge [Fabaceae; astragali radix praeparata cum mell], Panax ginseng C.A.Mey. [Araliaceae; folium ginseng], Descurainia sophia L.) Webb ex Prantl [Brassicaceae; lepidii semen], Leonurus japonicus Houtt. [Lamiaceae; extractum leonuri inspissatum], Panax notoginseng (Burkill) F.H.Chen [Araliaceae; notoginseng radix et rhizoma]*	NA	Prepared by the Department of Pharmaceutics, Affiliated Hospital of Liaoning University of Traditional Chinese Medicine.	SD rats	Coronary artery coarctation method	9.2 g,10 mL	1.BNP↓.5.mtTFA mRNA↑, mtTFA↑.	①⑤	Ning (2019)
3	Yiqi Huoxue decoction	*SCodonopsis pilosula (Franch.) Nannf. [Campanulaceae; codonopsis radix], Astragalus mongholicus Bunge [Fabaceae; astragali radix praeparata cum mell], Salvia miltiorrhiza Bunge [Lamiaceae; salviae miltiorrhizae radix et rhizoma], Panax notoginseng (Burkill) F.H.Chen [Araliaceae; notoginseng radix et rhizoma]*	17:33:33:11	Prepared by Liu. (2017) according to China Pharmacopoeia.	Wistarmale rats	Abdominal aorta coarctation method	8.5 g,10 mL	1.LVAWd + LVPWd↓, E/e'↓, LVEDP↓2.LW↓.3.HW↓.5.PARmax↓, CaMKII↓, PKA↓, NCX1/SERCA2a↓, PLB(S16)↑, RyR2↓.6.FIB↓.	①②③⑤⑥	Liu (2017)
4	Yiqi Huoxue decoction	*Astragalus mongholicus Bunge [Fabaceae; astragali radix praeparata cum mell], SCodonopsis pilosula (Franch.) Nannf. [Campanulaceae; codonopsis radix], Salvia miltiorrhiza Bunge [Lamiaceae; salviae miltiorrhizae radix et rhizoma], Panax notoginseng (Burkill) F.H.Chen [Araliaceae; notoginseng radix et rhizoma]*	33:17:17:17	Prepared by the preparation Room of Dongfang Hospital, Beijing University of Chinese Medicine (Tongrentang).	SD rats	Abdominal aorta coarctation method	7.5 g,10 mL	1.LVAWd + LVPWd↓, E/A↑, IVRT↓.5.CaMKIIδB↓, CaMKIIδC↓, SERCA2a↑.	①⑤	Chen (2014)
5	Yangxin No.2 granules	*Pseudostellaria heterophylla (Miq.) Pax [Caryophyllaceae; pseudostellariae radix], Astragalus mongholicus Bunge [Fabaceae; astragali radix praeparata cum mell], Salvia miltiorrhiza Bunge [Lamiaceae; salviae miltiorrhizae radix et rhizoma], Polyporus umbellatus (Pers.) Fr. [Polyporaceae; Grifola umbellataPilat], Conioselinum anthriscoides ‘Chuanxiong’ [Apiaceae; chuanxiong rhizoma], SCodonopsis pilosula (Franch.) Nannf. [Campanulaceae; codonopsis radix], Schisandra chinensis (Turcz.) Baill. [Schisandraceae; fructus schisandrae chinensis], Leonurus japonicus Houtt. [Lamiaceae; extractum leonuri inspissatum], Ophiopogon japonicus (Thunb.) Ker Gawl. [Asparagaceae; ophiopogonis radix], Polygonatum kingianum Collett and Hemsl. [Asparagaceae; polygonati rhizoma], Steleophaga plancyi (Boleny) [Blattidae; eupolyphaga steleophaga]*	NA	Produced by Jiangyin Tianjiang Pharmaceutical Co., LTD.	SD rats	Isoproterenol intraperitoneal injection	3.24g, 9 mL	1.LVEF↑, LVFS↑.4.TNF-α↓, IL一1β↓, IL-6↓, IL-10↑.	①④	Tang (2016)
6	Xinjierkang oral liquid	*Panax ginseng C.A.Mey. [Araliaceae; ginseng folium], Polygonatum odoratum (Mill.) Druce [Asparagaceae; polygonati odorati rhizoma], Panax notoginseng (Burkill) F.H.Chen [Araliaceae; notoginseng radix et rhizoma], Allium chinense G.Don [Amaryllidaceae; allii macrostemonis bulbus], Angelica sinensis (Oliv.) Diels [Apiaceae; angelicae sinensis radix], Ophiopogon japonicus (Thunb.) Ker Gawl. [Asparagaceae; ophiopogonis radix], Schisandra chinensis (Turcz.) Baill. [Schisandraceae; schisandrae chinensis fructus], Salvia miltiorrhiza Bunge [Lamiaceae; salviae miltiorrhizae radix et rhizoma], Sophora flavescens Aiton [Fabaceae; sophorae flavescentis radix], Glycyrrhiza glabra L. [Fabaceae; glycyrrhizae radix et rhizoma], Astragalus mongholicus Bunge [Fabaceae; astragali radix], Epimedium brevicornu Maxim. [Berberidaceae; Pharmaceutical], Trichosanthes kirilowii Maxim. [Cucurbitaceae; trichosanthis semen tostum], Blumea balsamifera L.) DC. [Asteraceae; l-borneolum]*	11.71:7.03:3.09:7.80:7.80:7.80:3.93:7.80:7.80:7.80:11.69:7.80:7.80:0.15	Purchased from Hefei Company of Traditional Crude Drugs.	Wistarmale male rats	Clipping right renal artery	6,12,24 g	1.LVSP↓, +dP/dtmax↓.3.SBP↓, HW/BW↓.4.Cardiac myocytes (CsA,CVF,PVCA)↓, SOD↑, MDA↓.6.NO↑, AngII↓.	①③④⑥	Yu et al. (2013)
7	Xindening oral liquid	*Panax ginseng C.A.Mey. [Araliaceae; ginseng folium], Salvia miltiorrhiza Bunge [Lamiaceae; salviae miltiorrhizae radix et rhizoma], Allium chinense G.Don [Amaryllidaceae; allii macrostemonis bulbus], Ephedra sinica Stapf [Ephedraceae; ephedrae radix et rhizoma], Wolfiporia cocos (Schw.) Ryv. and Cilbn. [Polyporaceae],*	NA	Prepared by the 152nd Hospital of PLA, Pingdingshan, China.	Wistarmale male rats	TAC	5,10,20 mL	1.LVEDP↓, LVSP↑, dp/dtmax↑, LVMI↓, BNP↓.3.HW↓, HR↑.4.LDH↓, cTnI↓, CK-MB↓, collagen fiber↓.6.TGF-β1↓, Smad3↓, p38 MAPK↓.	①③④⑥	Wei et al. (2017)
8	Xiaoqinglong decoction	*Ephedra sinica Stapf [Ephedraceae; ephedrae radix et rhizoma], Paeonia lactiflora Pall. [Paeoniaceae; paeoniae radix alba], Asarum heterotropoides F.Schmidt [Aristolochiaceae; asari radix et rhizoma], Zingiber officinale Roscoe [Zingiberaceae; extractum zingiberis liquidum], Glycyrrhiza glabra L. [Fabaceae; extractum glycyrrhizae liquidum], Neolitsea cassia L.) Kosterm. [Lauraceae; cinnamomi cortex], Schisandra sphenanthera Rehder and E.H.Wilson [Schisandraceae; schisandrae sphenantherae fructus], Pinellia ternata (Thunb.) Makino [Araceae; pinelliae rhizoma]*	3:3:1:2:1:2:3	Purchased from the Affiliated Hospital of Shandong University of Traditional Chinese Medicine.	Dahl salt-sensitive rats	8% NaCl high salt diet	4.0 g	1.LVIDd↓, LVEF↑, NT-proBNP↓.3.LVWT↓.4.CollagenI and CollagenIII↓, MCP-1↓, TNF-α↓, IL6↓.	①③④	Zhou (2020)
9	Xiaoqinglong decoction	*Ephedra sinica Stapf [Ephedraceae; ephedrae radix et rhizoma], Paeonia lactiflora Pall. [Paeoniaceae; paeoniae radix alba], Asarum heterotropoides F.Schmidt [Aristolochiaceae; asari radix et rhizoma], Zingiber officinale Roscoe [Zingiberaceae; extractum zingiberis liquidum], Glycyrrhiza glabra L. [Fabaceae; extractum glycyrrhizae liquidum], Neolitsea cassia L.) Kosterm. [Lauraceae; cinnamomi cortex], Schisandra sphenanthera Rehder and E.H.Wilson [Schisandraceae; schisandrae sphenantherae fructus], Pinellia ternata (Thunb.) Makino [Araceae; pinelliae rhizoma]*	3:1:2:1:2:1:3	Provided by the Affiliated Hospital of Shandong University of Traditional Chinese Medicine.	Dahl salt-sensitive male rats	8% NaCl high salt diet	4.0g, 2 mL	1.LVDd↓, LVEF↑, SBP↓, NT-proBNP↓.3.BW↑, LVWT↓, LVM↓.4.CollagenI and collagenIII↓, TNF-α↓, IL-6 ↓, ZO-1mRNA↑, serum endotoxin↓, acetate↑, propionate↑, butyrate↑.6.Fibrosis of the left ventricle↓.	①③④⑥	Zhou et al. (2019)
10	Tongmai Yangxin pill	*Rehmannia glutinosa (Gaertn.) DC. [Orobanchaceae; radix rehmanniae praeparata], Ophiopogon japonicus (Thunb.) Ker Gawl. [Asparagaceae;ophiopogonis radix], Glycyrrhiza glabra L. [Fabaceae; extractum glycyrrhizae liquidum], Reynoutria multiflora (Thunb.) Moldenke [Polygonaceae; polygoni multiflori caulis], Asarum heterotropoides F.Schmidt [Aristolochiaceae;radix et rhizoma asari], Neolitsea cassia L.) Kosterm. [Lauraceae; cinnamomi ramulus]*	NA	Provided by Tianjin Zhongxin Pharmaceutical Group Co., Ltd.	SD rats	Coronary artery coarctation method	4.0 g	1.LVIDd↓, LVIDs↓, LVPWs↑, LVEF↑, LVFS↑.3.LVM↓.4.CK↓, CK-MB↓, LDH↓, MDA↓, Gs-α↑, AC proteins↑, PKA gene and protein↑, ROCK gene and protein↓.6.NO↑, the expression and phosphorylation of eNOS↑.	①③④⑥	Chen et al. (2021)
11	Songling Xuemaikang capsule	*Pueraria montana* var. *lobata (Willd.) Maesen and S.M.Almeida ex Sanjappa and Predeep [Fabaceae; puerariae lobatae radix], Pinus massoniana Lamb. [Pinaceae; pini lignum nodi], Fritillaria thunbergii Miq.[Liliaceae; fritillariae thunbergii bulbus]*	NA	Provided by KangHong Pharmaceutical Group.	SD rats	Isoproterenol intraperitoneal injection	3.24 g,1 mL	3.LVPWd↓, HW↓, HW/BW↓, BNP↓.5.The phosphorylations of ERK1/2↓, CaMKIIδ↓, GATA4↓.	③⑤	Qi et al. (2020)
12	Sini decoction	*Aconitum carmichaelii Debeaux [Ranunculaceae; aconiti lateralis radix praeparata], Glycyrrhiza glabra L.[Fabaceae; extractum glycyrrhizae liquidum], Zingiber officinale Roscoe [Zingiberaceae; extractum zingiberis liquidum]*	7.5:10:15	Purchased from the First Affiliated Hospital of Hunan University of Chinese Medicine.	SD rats	Adriamycin intraperitoneal injection	2.8 g,2.8 mL	1.LVEF↑, LVFS↑, NT-proBNP↓.4.endotoxin LPS↓, OTUS↓.	①④	Zhao et al. (2022)
13	Simiao Yongan decoction	*Lonicera japonica Thunb. [Caprifoliaceae; caulis lonicerae japonicae], Scrophularia ningpoensis Hemsl. [Scrophulariaceae; scrophulariae radix], Angelica sinensis (Oliv.) Diels [Apiaceae; angelicae sinensis radix], Glycyrrhiza glabra L. [Fabaceae; extractum glycyrrhizae liquidum]*	3:3:2:1	Prepared by Li et al. (2020) according to China Pharmacopoeia.	C57BL/6J male mice	STZ intraperitoneal injection	12.29 g,8.2 mL	1.LVEF↑, LVFS↑, E/A↑.4.CollagenI and CollagenIII↓, CD45↓, NF-κB-p65↓.5.Caspase-3↓, TC↓, LDL-C↓, NEFA↓, GLC↓, GCGR↓, PGC-1α↓, PPARα↓, p-AMPK↓, GLU↓.6.MAPK.	①④⑤⑥	Li et al. (2020)
14	Shunxin decoction	*Astragalus mongholicus Bunge [Fabaceae; astragali radix praeparata cum mell], Panax ginseng C.A.Mey. [Araliaceae; ginseng folium], Atractylodes macrocephala Koidz. [Asteraceae; atractylodis macrocephalae rhizoma], Cichorium intybus L. [Asteraceae; cichorii herba, cichorii radix], Wolfiporia cocos (Schw.) Ryv. and Cilbn. [Polyporaceae], Salvia miltiorrhiza Bunge [Lamiaceae; salviae miltiorrhizae radix et rhizoma], Conioselinum anthriscoides ‘Chuanxiong’ [Apiaceae; chuanxiong rhizoma], Paeonia anomala subsp. veitchii (Lynch) D.Y.Hong and K.Y.Pan [Paeoniaceae; paeoniae radix rubra], Glycyrrhiza glabra L. [Fabaceae; glycyrrhizae radix et rhizoma]*	NA	Purchased from the Hui Ren Tang Pharmaceutical Company	SD rats	Abdominal aorta coarctation method	14.27, 42.81 g	1.IVSd↓, PWd↓, E/A↓, NT-proBNP↓.3.CVF↓.4.myocardial GC↑, myocardial NPRA↑.5.Volume of mitochondria↓.6.eNOS↑, PDE5A↓, PKG I↑, cGMP↑.	①③④⑤⑥	Jia et al. (2022)
15	Shensong Yangxin	*Panax ginseng C.A.Mey. [Araliaceae; folium ginseng], Ophiopogon japonicus (Thunb.) Ker Gawl. [Asparagaceae; ophiopogonis radix], Cornus officinalis Siebold and Zucc. [Cornaceae; corni fructus], Salvia miltiorrhiza Bunge [Lamiaceae; salviae miltiorrhizae radix et rhizoma], Paeonia anomala subsp. veitchii (Lynch) D.Y.Hong and K.Y.Pan [Paeoniaceae; paeoniae radix rubra], Ziziphus jujuba Mill. [Rhamnaceae; jujubae fructus], Taxillus chinensis (DC.) Danser [Loranthaceae; taxilli herba], Paeonia anomala subsp. veitchii (Lynch) D.Y.Hong and K.Y.Pan [Paeoniaceae; paeoniae radix rubra], Steleophaga plancyi (Boleny) [Blattidae; eupolyphaga steleophaga], Nardostachys jatamansi (D.Don) DC. [Caprifoliaceae; nardostachyos radix et rhizoma], Coptis chinensis Franch. [Ranunculaceae; coptidis rhizoma], Schisandra chinensis (Turcz.) Baill. [Schisandraceae; schisandrae chinensis fructus], Fossilia Ossis Mastodi (Baill.) [Fabaceae; Os Draconis]*	NA	Purchased from Shijiazhuang Yiling Pharmaceutica.	SD rats	Abdominal aorta coarctation method	0.2, 0.8, 4 g	1.LVAWd + LVPWd↓, LVIDd↓, LVEF↑, LVFS↑, NT-proBNP↓.3.HW↓, LVM↓, HR↓.4.PKA↓5.CaMKII↓, RyR2↓, SERCA2a↑.	①③④⑤	Zhang et al. (2018)
16	Shenqi Lixin Decoction	*Panax ginseng C.A.Mey. [Araliaceae; folium ginseng], Astragalus mongholicus Bunge [Fabaceae; astragali radix praeparata cum mell], Neolitsea cassia L.) Kosterm. [Lauraceae; cinnamomi cortex], Epimedium brevicornu Maxim. [Berberidaceae; epimedii folium], Senna obtusifolia L.) H.S.Irwin and Barneby [Fabaceae; cassiae semen], Angelica biserrata (R.H.Shan and C.Q.Yuan) C.Q.Yuan and R.H.Shan [Apiaceae; angelicae pubescentis radix], Salvia miltiorrhiza Bunge [Lamiaceae; salviae miltiorrhizae radix et rhizoma], Wolfiporia cocos (Schw.) Ryv. and Cilbn. [Polyporaceae], Atractylodes macrocephala Koidz. [Asteraceae; atractylodis macrocephalae rhizoma], Agrimonia pilosa Ledeb. [Rosaceae; agrimoniae herba], Leonurus japonicus Houtt. [Lamiaceae; leonuri fructus], Glycyrrhiza glabra L. [Fabaceae; glycyrrhizae radix et rhizoma]*	20:20:10:20:15:15:20:15:30:15:10	Purchased from Shanghai Slack Laboratory Animal Co., Ltd.	Wistarmale male rats	Adriamycin intraperitoneal injection	9.975, 19.95, 39.90g10 mL	1.NT-proBNP↓,4.Myocardial tissue level of ATP↓,CMAR↓5.Bcl-2mRNA and PGC-1αmRNA↑, BaxmRNA and Caspase-3 mRNA↓,Bcl2↑,Bax↓,Caspase-3↓,P53↓.	①④⑤	Zhuang et al. (2021)
17	Shengmai decoction	*Panax ginseng C.A.Mey. [Araliaceae; folium ginseng], Ophiopogon japonicus (Thunb.) Ker Gawl. [Asparagaceae; ophiopogonis radix], Schisandra chinensis (Turcz.) Baill. [Schisandraceae; schisandrae chinensis fructus]*	1:2:1	Purchased from Jilin Tianqiang Pharmaceutical Co., LTD.	SD rats	STZ intraperitoneal injection and high fat diet	1.05 g	1.LVEF↑,LVFS↑,LVEDP↓.3.HMI↓.5.SERCA2a↑,PLBser16↑,NCX1↑,CaMKII↓.	①③⑤	Zhai (2020)
18	Shengmai decoction	*Panax ginseng C.A.Mey. [Araliaceae; folium ginseng], Ophiopogon japonicus (Thunb.) Ker Gawl. [Asparagaceae; ophiopogonis radix], Schisandra chinensis (Turcz.) Baill. [Schisandraceae; schisandrae chinensis fructus]*	10:15:06	Purchased from Tauto Biotech Company.	BKSCg-m+/+LeprdbNJU mice (db/db mice)	Unreported	4.5 g,5.6 mL	3.HW↓, -dp/dtmax↑.6.MMP2↑, MMP9↑, TIMP-2↓.	③⑥	Zhao et al. (2016)
19	Shenfu Xiongze decoction	*Astragalus mongholicus Bunge [Fabaceae; astragali radix praeparata cum mell], Panax ginseng C.A.Mey. [Araliaceae; folium ginseng], Aconitum carmichaelii Debeaux [Ranunculaceae; aconiti lateralis radix praeparata], Salvia miltiorrhiza Bunge [Lamiaceae; salviae miltiorrhizae radix et rhizoma], Alisma plantago-aquatica subsp. orientale (Sam.) Sam. [Alismataceae; alismatis rhizoma], Conioselinum anthriscoides ‘Chuanxiong’ [Apiaceae; chuanxiong rhizoma]*	30:10:6:9:15:30	Purchased from Guangdong Yifang Pharmaceutical Co., LTD.	Dahl salt-sensitive male rats	8% NaCl high salt diet	6.4 g,10 mL	1.LVEF↑, BNP↓, NT-ProBNP↓.3.BW↑.4.vWF↓, PECAM-1(CD31)↓, cdc42↓.6.p38↓, MAPK/APK↓, HSP27↓.	①③④⑥	Zhang (2020)
20	Shenfu decoction	*Panax ginseng C.A.Mey. [Araliaceae; folium ginseng], Aconitum carmichaelii Debeaux [Ranunculaceae; aconiti lateralis radix praeparata]*	NA	Prepared by Huang. (2019) according to China Pharmacopoeia.	C57BL/6 male mice	TAC	0.2003g	1.LVEF↑,LVFS↑,BNP↓.2.LW/BW↓.3.HW/BW↓,HW/TL↓,ANP↓.4.CollagenI and CollagenIII↓, ST2↓.5.PGC-1α↑, PGC-1β↑, ERRα↑, ERRβ↑, CPT-1A↑, PDK4↑, CD36↑, GLUT4↑.6.SMA ↑, α-MHC↑, Fibronectin↓, β-MHC↓MMP-9↓, MMP-2↓.	①②③④⑤⑥	Huang (2019)
21	Qishen Yiqi decoction	*Astragalus mongholicus Bunge [Fabaceae; astragali radix praeparata cum mell], Salvia miltiorrhiza Bunge [Lamiaceae; salviae miltiorrhizae radix et rhizoma], Panax notoginseng (Burkill) F.H.Chen [Araliaceae; notoginseng radix et rhizoma], Dalbergia odorifera T.C.Chen [Fabaceae; dalbergiae odoriferae lignum]*	10:5:1:0.067	Purchased from Tasly Pharmaceutical Group Co., Ltd	C57BL/6 N mice	Infusing L-NAME and feeding a high fat diet	0.58, 1.16 g	1.LVPW↓, LA/Ao↓, E/A↑, IVCT↓, BNP↓.3.HW/TL↓, LVM↓, IVS↓, SBP↓, ANP↓.4.CollagenI and collagenIII↓, TNF-α↓, MCP-1↓, ROS↓, NF-κB↓, NLRP3↓, iNOS↓, ICAM-1, VCAM-1, Eselectin↓, EndMT markers (SM22α,TWIST1, and Calponin)↑, NOX2 and NOX3↓.6.NO↑, cGMP↑, PKG↑, titin↓.	①③④⑥	Huang et al. (2021)
22	Poge Heart-Saving decoction	*Aconitum carmichaelii Debeaux [Ranunculaceae; aconiti lateralis radix praeparata], Glycyrrhiza glabra L. [Fabaceae; glycyrrhizae radix et rhizoma], Zingiber officinale Roscoe [Zingiberaceae;extractum zingiberis liquidum], Cornus officinalis Siebold and Zucc. [Cornaceae; corni fructus], Fossilia Ossis Mastodi (Baill.) [Fabaceae; Os Draconis], Crassostrea gigas (Thunberg) [Ostreidae; Ostreae Testa], Magnetite (Lodestone) L. [Spinel; Magnetitum] Panax ginseng C.A.Mey.[Araliaceae; folium ginseng], Abelmoschus moschatus Medik. [Malvaceae; ambrette]*	150:60:60:120:30:30:30:30:0.5	Prepared by Liu et al. (2020) according to China Pharmacopoeia.	SD rats	Adriamycin intraperitoneal injection	9.33, 13.995, 18.66g10 mL	1.LVEDs↓, LVEF↑.4.serum ALD↓.6.AngII↓.	①④⑥	Liu et al. (2020)
23	Nuanxin capsule	*Panax ginseng C.A.Mey. [Araliaceae; ginseng folium], Aconitum carmichaelii Debeaux [Ranunculaceae; aconiti lateralis radix praeparata], Coix lacryma-jobi* var. *ma-yuen (Rom.Caill.) Stapf [Poaceae; coicis semen], Citrus × aurantium f. deliciosa (Ten.) M.Hiroe [Rutaceae; citri exocarpium rubrum]*	NA	Prepared by the Pharmaceutical Products Room of Guangdong Provincial Hospital of Traditional Chinese Medicine.	C57BL/6J mice	TAC	0.64, 1.72 g	1.LVIDd↓, LVIDs↓, LVEF↑, LVFS↑, BNP↓.4.ROS↓.5.OCR↑, p-AMPK/AMPK↑, PGC-1α/GAPDH↑, mitochondria ATP↑.	①④⑤	Lou (2021)
24	Linggui Qihua decoction	*Cichorium intybus L. [Asteraceae; cichorii herba, cichorii radix], Atractylodes macrocephala Koidz. [Asteraceae; atractylodis macrocephalae rhizoma], Neolitsea cassia L.) Kosterm. [Lauraceae; cinnamomi cortex], Paeonia anomala subsp. veitchii (Lynch) D.Y.Hong and K.Y.Pan [Paeoniaceae; paeoniae radix rubra]*	NA	Provided by the Department of Pharmaceutical Products, Xiyuan Hospital, China Academy of Chinese Medical Sciences.	SHR rats	STZ intraperitoneal injection and high fat diet	4.05, 8.10 g	1.E↓, E/A↓, E/e'↓, BNP↓.3.ANP↓.4.hs-CRP↓.5.FINS↓, C-P↓, LEP↓, GSP↓, GLU↓.	①③④⑤	Xiong et al. (2021)
25	Linggui Qihua decoction	*Wolfiporia cocos (Schw.) Ryv. and Cilbn. [Polyporaceae], Atractylodes macrocephala Koidz. [Asteraceae; atractylodis macrocephalae rhizoma], Neolitsea cassia L.) Kosterm. [Lauraceae; cinnamomi cortex], Paeonia anomala subsp. veitchii (Lynch) D.Y.Hong and K.Y.Pan [Paeoniaceae; paeoniae radix rubra]*	NA	Provided by the Department of Pharmaceutical Products, Xiyuan Hospital, China Academy of Chinese Medical Sciences.	SHR rats	STZ intraperitoneal injection and high fat diet	4.05, 8.10 g	1.E/A↓, LA (DI/R)↓, BNP↓.2.RA (DI/R)↓.3.ANP↓.4.hs-CRP↓, FINS↓, C-P↓, GSP↓, GLU↓, TC↓, LDL-CHO↓, AT1 mRNA↓.6.TGF-β1 mRNA↓, TIMP-1 mRNA↓, Smad3 mRNA↓, MMP-9mRNA↑.	①②③④⑥	Xiong (2022)
26	Kangdaxin oral liquid	*Aconitum carmichaelii Debeaux [Ranunculaceae; aconiti radix cocta], Astragalus mongholicus Bunge [Fabaceae; astragali radix praeparata cum mell], Santalum album L. [Santalaceae; santali albi lignum]*	NA	Prepared by People’s Hospital Affiliated to Fujian University of Traditional Chinese Medicine.	SD rats	Abdominal aorta coarctation method	2.7 mL	1.E/e'↓, E/A↑, NT-proBNP↓.3.HW/BW↓, LVw/W↓.6.Myocardial fibrosis↓, TGF-β1↓.	①③⑥	Wu et al. (2022)
27	Jiawei Shenfu granules	*Panax ginseng C.A.Mey. [Araliaceae; ginseng folium], Aconitum carmichaelii Debeaux [Ranunculaceae; aconiti lateralis radix praeparata], Descurainia sophia L.) Webb ex Prantl [Brassicaceae; descurainiae semen, lepidii semen], Plantago asiatica L. [Plantaginaceae; plantaginis semen], Panax notoginseng (Burkill) F.H.Chen [Araliaceae; notoginseng radix et rhizoma]*	NA	Prepared by Guangdong Yifang Pharmaceutical Factory	SD rats	Abdominal aorta coarctation method	6.84, 13.68 g	1.HR↓, ±dP/dt max↑. 3.SAP↓, DAP↓, LVEDP↓.5.Na + -K + -ATP enzyme↑, Ca2+-ATP enzyme↑.	①③⑤	Liu (2013)
28	Gegen Qinlian decoction	*Pueraria montana* var. *lobata (Willd.) Maesen and S.M.Almeida ex Sanjappa and Predeep [Fabaceae; puerariae lobatae radix], Scutellaria baicalensis Georgi [Lamiaceae; extractum scutellariae], Coptis chinensis Franch. [Ranunculaceae; coptidis rhizoma], Glycyrrhiza glabra L. [Fabaceae; glycyrrhizae radix et rhizoma]*	5:3:3:2	Prepared by Han et al. (2017) according to China Pharmacopoeia.	C57BL/KsJ male mice (db/db mice)	Unreported	23.4 g	1.E/A↑.5.GLU↓, Acer2↑, Slc38a2↑, Ppp1r3c↑, PGam1↑, Ceramide↓, Pyruvate↑.	①⑤	Han et al. (2017)
29	Fuzi Banxia decoction	*Aconitum carmichaelii Debeaux [Ranunculaceae; aconiti lateralis radix praeparata], Pinellia ternata (Thunb.) Makino [Araceae; pinelliae rhizoma]*	NA	Purchased from Beijing Huamiao Pharmaceutical Co. Ltd.	SD rats	Abdominal aorta coarctation method	10.8 g	1.LVEF↑, BNP.3.LVM↓, cTnI↓.4.Collagen deposition↓, PKA↓.6.pSer346↓, pSer(355,356)↓, GRK2↓.	①③④⑥	Sun et al. (2019)
30	Buyang Huanwu decoction	*Astragalus mongholicus Bunge [Fabaceae; astragali radix praeparata cum mell], Angelica sinensis (Oliv.) Diels [Apiaceae; angelicae sinensis radix], Paeonia anomala subsp. veitchii (Lynch) D.Y.Hong and K.Y.Pan [Paeoniaceae; paeoniae radix rubra], Pheretima guillelmi (Michaelsen) [Megascolecidae; Lumbricus], Conioselinum anthriscoides ‘Chuanxiong’ [Apiaceae; chuanxiong rhizoma], Prunus persica L.) Batsch [Rosaceae; persicae semen], Carthamus tinctorius L. [Asteraceae; carthami flos]*	120:6:4.5:3:3:3:3	Purchased from Beijing Tongrentang Technology Development Co., LTD.。	SD rats	Abdominal aorta coarctation method	12.72 g	1.LVEDP↓, ventricular muscle tone↓.	①	Dong et al. (2018)

①Improve left heart congestion, adjust diastolic dysfunction, and prevent the left atrial hypertension.②Regulate pulmonary vascular disease and right ventricular dysfunction.③Ameliorate plasma volume expansion.④Inhibit systemic microvascular inflammation.

#### 4.2.1 Traditional dosage form

##### 4.2.1.1 Decoction

As the most important method of clinical prevention and treatment of diseases in TCM, traditional Chinese medicine decoctions have the characteristics of individual prescription, adding and decreasing with the syndrome, and customizing measures to personal conditions (Chou et al., 2022). At present, it is known that traditional Chinese medicine decoctions can improve clinical symptoms, adjust patients’ cardiac function and prognosis, improve quality of life, regulate abnormal biomarkers, and so on. Most of the decoctions in the study can reduce the TCMSS or TCMFDIS score. Yiqi Yangyin Huoxue decoction (Shen, 2018), Danting Lishui decoction (Chen, 2014; Zou et al., 2014; Zou et al., 2014), Shengui Huxin Huacai decoction (Zhu, 2017), Yiqi Yangyin decoction (Zhang, 2018), Xinkang decoction (Chen, 2019), Shuxin Huoxue decoction (Ma et al., 2020), Guiqi Huxin decoction (Li, 2020), Xiaoqinglong decoction (Zhou, 2020), Shenqi Yixin Huacai decoction (Zhang, 2020), Yiqi Tongyu decoction (Li, 2021), Yiqi Huoxue2 decoction (Liu et al., 2021), Danling Huoxue Huayin decoction (Ji, 2021), Baoyuan Yangxin decoction (Wang, 2021), Tiaogan Yangxin decoction (Sun, 2021) and Modified Shenqi Yixin decoction (Liu et al., 2021) can clearly reduce the clinical symptoms and improve the curative effect of TCM syndrome. Xinkan*g* decoction (Chen and Ye, 2019), Shuxin Huoxue decoction (Ma et al., 2020), Guiqi Huxin decoction (Li, 2020), Danling Huoxue Huayin decoction (Ji, 2021), Yiqi Tongyu decoction (Li, 2021), Yiqi Huoxue2 decoction (Liu et al., 2021), Baoyuan Yangxin decoction (Wang, 2021), Modified Shenqi Yixin decoction (Liu et al., 2021), Huoxue Lishui decoction (Zhou and Hong, 2016), Huangqi Baoxin decoction (Wu, 2018), Yangyin Shuxin decoction (Liu, 2020; Zhao, 2021; Li et al., 2022), Yiqi Huoxue Lishui decoction (Peng et al., 2020), Shengmai powder and Guizhi Gancao Longgu Muli decoction (Yuan, 2021) can increase the 6-min walking distance, improve cardiopulmonary motor function, and enhance the overall motor function of patients. Yiqi Yangyin Huoxue decoction (Shen, 2018), Yiqi Yangyin decoction (Zhang, 2018), Xinkang decoction (Chen and Ye, 2019), Danling Huoxue Huayin decoction (Ji, 2021), Yiqi Tongyu decoction (Li, 2021), Yangyin Shuxin decoction (Liu, 2020), Shengmai powder and Guizhi Gancao Longgu Muli decoction (Yuan, 2021), Qiangxin Tongmai decoction (Zhang, 2019) and Yiqi Huoxue1 decoction (Liu et al., 2020) can reduce the MLHFQ score and improve quality of life. Tiaogan Yangxin decoction (Sun, 2021), Qiangxin Tongmai decoction (Zhang, 2019), and Yiqi Huoxue1 decoction (Liu et al., 2020) can reduce SDS or SAS scores and improve anxiety and depression symptoms. Danting Lishui decoction (Zou et al., 2014), Danling Huoxue Huayin decoction (Ji, 2021), and Baoyuan Yangxin decoction (Wang, 2021) can improve NT-proBNP, prevent the development of HFpEF, improve prognosis, and reduce mortality and rehospitalization rates. Shuxin Huoxue decoction (Ma et al., 2020), Xiaoqinglong decoction (Zhou, 2020), Tiaogan Yangxin decoction (Sun, 2021), and Huoxue Lishui decoction (Zhou and Hong, 2016) can increase the cardiac ejection fraction and improve cardiac function. Huoxue Lishui decoction (Zhou and Hong, 2016) can reduce hs-CRP, improve inflammation, and maintain intravascular homeostasis. Danting Lishui decoction (Zou et al., 2014) can reduce FINS and Homa-IR and interfere with insulin resistance. Guiqi Huxin decoction (Li, 2020) can increase oestradiol, protect the heart, and improve endocrine metabolism. Baoyuan Yangxin decoction (Wang, 2021) reduces TGF-β1 and controls myocardial fibrosis and left ventricular hypertrophy. Danting Lishui decoction (Chen, 2014; Zou et al., 2014), Shengui Huxin Huacai decoction (Zhu, 2017), Baoyuan Yangxin decoction (Wang, 2021), Tiaogan Yangxin decoction (Sun, 2021), and Modified Shenqi Yixin decoction (Liu et al., 2021) can decrease the parameters of E/e’ and LAVI to improve left ventricular diastolic function and inhibit left ventricular remodelling. Yangyin Shuxin decoction (Li et al., 2020) can enhance cardiac pumping function, reduce myocardial oxygen consumption, and improve coronary artery circulation.

##### 4.2.1.2 Plaster

Traditional Chinese medicine plasters are divided into edible black plasters and external applications of white plasters. This article only considers the black plaster prescriptions. Compared with decoctions, black plaster prescriptions can reduce gastrointestinal reactions, enhance the safety of drug use, and help to improve the compliance of patients (Jiang and Tong, 2022). Yangxin Huoxue Tongmai plaster (Wang, 2019; Li, 2020) and Shenqi Baoxin plaster (Chen, 2019) can improve TCM clinical symptoms, enhance TCM efficacy, increase 6-min walking distance, enhance exercise endurance, improve NT-proBNP, prevent the development of HFpEF, improve prognosis, and reduce mortality and rehospitalization rates. Shenqi Baoxin plaster (Chen, 2019) can also reduce MLHFQ scores and improve quality of life.

#### 4.2.2 Modern dosage form

##### 4.2.2.1 Granules

The main advantage of granules is that they have the same curative effect as a decoction, and they are easy to carry and take orally; thus, they are widely used in TCM clinics (Qin et al., 2022). Qifu Qiangxin granules (Bai, 2022), Yangyin Shuxin granules (Zhao et al., 2018), Wenyang Huoxue granules (Gu, 2016), Bawei Tongluo granules (Zhang, 2017), Xinshuaining Heji granules (Wan, 2017), Shuxin granules (Chen, 2018), Jiangzhuo Tongluo granules (Yin et al., 2019), Qigui Yiqi Huoxue granules (Liu, 2020), Jiawei Linggui Zhugan granules (Wang, 2021), Qihong granules (Li, 2021), Zhigancao granules (Zhang et al., 2021), and Jianxin granules (Xin, 2015; Chen et al., 2022) can improve the clinical symptoms of TCM, reduce the scores of TCM syndrome, improve the prognosis, and reduce the mortality and rehospitalization rate. Qifu Qiangxin granules (Bai, 2022), Yangyin Shuxin granules (Zhao et al., 2018), Bawei Tongluo granules (Zhang, 2017), Shuxin granules (Chen, 2018), Jiangzhuo Tongluo granules (Yin et al., 2019), Jiawei Linggui Zhugan granules (Wang, 2021), Qihong granules (Li, 2021), Jianxin granules (Xin, 2015), and Linggui Zhugan granules (Niu, 2021) can lower the MLHFQ score and improve quality of life. Jianxin granules (Chen et al., 2022) can reduce CRP, IL-1β, and IL-6 inflammatory factors to improve inflammation, maintain intravascular homeostasis, reduce BNP to improve prognosis and reduce mortality and rehospitalization rates. Qifu Qiangxin granules (Bai, 2022), Yangyin Shuxin granules (Zhao et al., 2018), Wenyang Huoxue granules (Gu, 2016), Xinshuaining Heji granules (Wan, 2017), Jiangzhuo Tongluo granules (Yin et al., 2019), Qigui Yiqi Huoxue granules (Liu, 2020), and Jianxin granules (Xin, 2015) can improve parameters such as E/e’, E/A, LVEF and LVMI to adjust the left ventricular diastolic function; enhance the cardiac pumping function; reduce myocardial oxygen consumption; inhibit the left ventricular remodelling; and improve coronary artery circulation. Yangyin Shuxin granules (Zhao et al., 2018), Wenyang Huoxue granules (Gu, 2016), Bawei Tongluo granules (Zhang, 2017), and Jiawei Linggui Zhugan granules (Wang, 2021) can increase the 6-min walking distance, enhance tolerance of daily activities and improve cardiopulmonary motor function. Yangyin Shuxin granules (Liu, 2020; Li et al., 2022) can increase the PeakVO index, |GLS| index, and UT index to regulate left ventricular systolic function and systolic synchronization and inhibit myocardial fibrosis and left ventricular hypertrophy.

##### 4.2.2.2 Capsule

TCM capsules are suitable for elderly patients and patients with diabetes. It has high bioavailability and can be rapidly disintegrated and absorbed into the intestines and stomach to achieve effective blood concentration (Zhang et al., 2012). Qili Qiangxin capsule (Lu et al., 2013), Tongmai Qiangxin capsule (Guan et al., 2015), Xinshuai Heji capsule (Gao et al., 2016), Qiangxin capsule (Wu, 2020), and Nuanxin capsule (Lou, 2021) can improve TCM symptoms and reduce the TCM syndrome score. Xinshuai Heji capsule (Gao et al., 2016) and Qiangxin capsule (Wu, 2020) can enhance tolerance of daily activities and improve cardiopulmonary motor function. Qili Qiangxin capsule (Lu et al., 2013), Tongmai Qiangxin capsule (Guan et al., 2015), Qiangxin capsule (Wu, 2020), and Nuanxin capsule (Lou, 2021) improve parameters such as E/e', E/A, LVEF and LAVI to adjust left ventricular systolic and diastolic function; inhibit left ventricular remodelling; enhance cardiac pumping function; reduce myocardial oxygen consumption; and improve coronary artery circulation. Qili Qiangxin capsule (Lu et al., 2013; Gao et al., 2019), Tongmai Qiangxin capsule (Guan et al., 2015), Xinshuai Heji capsule (Gao et al., 2016), and Nuanxin capsule (Lou, 2021) improve NT-proBNP, prevent the development of HFpEF, improve prognosis, and reduce mortality and rehospitalization rates.

In summary, the clinical evidence shows that the main clinical manifestations of TCM in the treatment of HFpEF are as follows: 1) improving the TCM symptoms and the exerting a curative effect on TCM syndrome; improving the quality of life, anxiety, depression, and fatigue of patients, including the MLHFQ score, SAS score, SDS score, and FS-14 score; 2) enhancing tolerance of daily activities and improving cardiopulmonary motor function, including NYHA grade, Lee’s heart failure score, 6-min walk test (6 MWT) and cardiopulmonary exercise test (CPET); 3) strengthening cardiac pumping function, improving systolic and diastolic function, reducing myocardial oxygen consumption, and improving coronary artery circulation. By improving parameters echocardiography such as E/e', E/A, LAVI, LVEF and LVMI, regulating left ventricular systolic function and systolic synchrony by adjusting STI parameters such as |GLS| and UT, and improving haemodynamics by increasing the CI and SI index; and 4) maintaining intravascular homeostasis, including reducing sST2 and hs-CRP to improve systemic inflammation; reducing CRP, IL-1β, IL-6, and other inflammatory factors and decreasing ET, TGF-β1, TNF-α, and AngII to control myocardial fibrosis and left ventricular hypertrophy; improving blood fat and blood pressure, such as total cholesterol (TC) and diastolic blood pressure; improving endocrine metabolism, including by increasing oestrogen E2 levels, interfering with insulin resistance, and reducing Homa-IR and FINS; reducing BNP and NT-proBNP to improve prognosis; and lowering mortality and rehospitalization rates, and preventing the development of HFpEF.

## 5 Potential mechanisms of TCM for HFpEF

**FIGURE 3:**
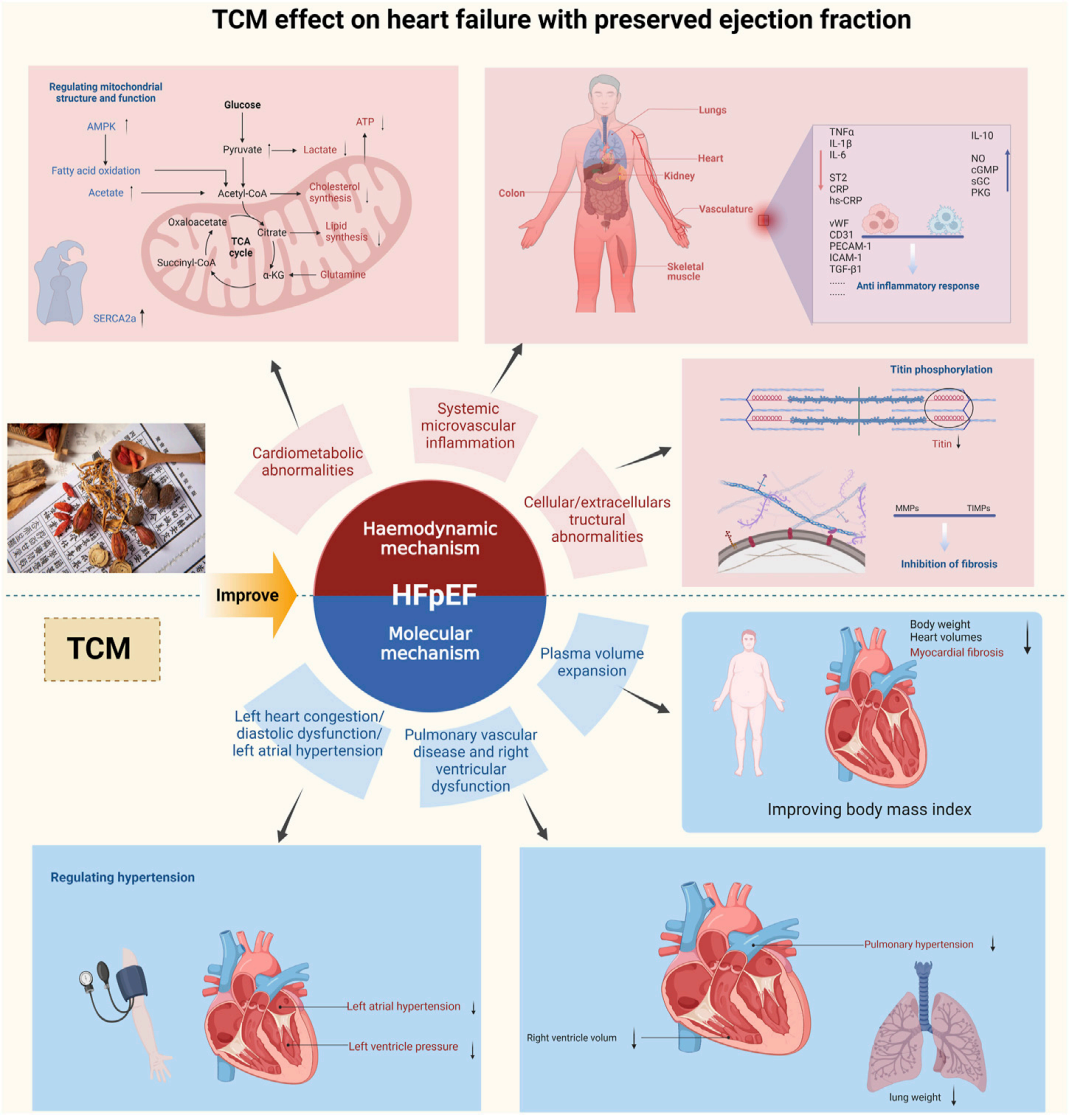
Potential mechanisms of TCM for HFpEF.

There is no unified conclusion on the specific mechanism of HFpEF. At present, it is widely believed that it is a multisystem disease involving the heart, lung, kidney, skeletal muscle, adipose tissue, immune/inflammatory signals, and vascular system (Mishra and Kass, 2021). According to haemodynamics and cellular and molecular mechanisms, HFpEF is divided into six types (Lam et al., 2018), as shown in Figure 3. There are few articles about the experimental types of HFpEF. In this paper, 12 articles about the experimental types of HFpEF were collected, and the other 18 articles were summarized concerning the similar mechanisms of haemodynamics or cellular molecular science in diastolic heart failure and chronic heart failure.

### 5.1 Haemodynamic mechanism

At present, three main haemodynamic mechanisms of HFpEF are observed according to the cardiac ultrasound parameters and the improvement of BNP and NT-proBNP.

#### 5.1.1 Left heart congestion/diastolic dysfunction/left atrial hypertension

As HFpEF was originally named after diastolic heart failure, most of the experimental studies in this paper have improved the problem of left ventricular diastolic dysfunction by regulating parameters such as E/e', E/A, LVEDP, BNP, and NT-proBNP. For example, Yiqihuoxue decoction (Chen, 2014; Liu, 2017) can improve diastolic function parameters by adjusting calcium ion homeostasis. Nuanxin capsule (Lou, 2021), Yangxin No. 2 granules (Tang, 2016), Shensong Yangxin decoction (Zhang et al., 2018), Fuzi Banxia decoction (Sun et al., 2019), Shenfu decoction (Huang, 2019), Xiaoqinglong decoction (Zhou et al., 2019; Zhou, 2020), Shengmai decoction (Zhai, 2020), Shenfu Xiongze decoction (Zhang, 2020), Simiao Yongan decoction (Li et al., 2020), Tongmai Yangxin pill (Chen et al., 2021), Sini decoction (Zhao et al., 2022), and Buyang Huanwu decoction (Dong et al., 2018) can improve left ventricular congestion and systolic function by regulating LVID d, LVID s, LVMI, LVEF, and LVFS. Qishen Yiqi decoction (Huang et al., 2021) and Linggui Qihua decoction (Xiong, 2022) can ameliorate left atrial hypertension by regulating LADI/R and LA/Ao.

#### 5.1.2 Pulmonary vascular disease and right ventricular dysfunction

Pulmonary vascular disease and right ventricular dysfunction are rare, the prognosis is poor, and the mortality is high (Lam et al., 2018). Shenfu decoction (Huang, 2019), Linggui Qihua decoction (Xiong, 2022), Yiqi Huoxue decoction (Liu, 2017), and Yiqi Xiefei decoction (Wang, 2019) can improve the lung weight index, lung weight, lung-to-body ratio, and corresponding ultrasound parameters. Among them, Linggui Qihua decoction (Xiong, 2022) can regulate the right atrial volume and reduce stress.

#### 5.1.3 Plasma volume expansion

The expansion of plasma volume is mainly related to right heart dilatation and total cardiac volume expansion. Shengmai decoction (Zhao et al., 2016; Zhai, 2020), Qishen Yiqi decoction (Huang et al., 2021), Linggui Qihua decoction (Xiong, 2022), Yiqi Huoxue decoction (Liu, 2017), Xinjierkang oral liquid (Yu et al., 2013), Xindening oral liquid (Wei et al., 2017), Shensong Yangxin Songling Xuemaikang capsule (Qi et al., 2020) and Kangdaxin oral liquid (Wu et al., 2022) can improve ANP, heart weight, the heart-to-body ratio, the myocardial ratio, and corresponding problems. Fuzi Banxia decoction (Sun et al., 2019), Xiaoqinglong decoction (Zhou et al., 2019; Zhou, 2020), Shenfu Xiongze decoction (Zhang, 2020), Sini decoction (Zhao et al., 2022), Qishen Yiqi decoction (Huang et al., 2021), Yiqi Xiefei decoction (Wang, 2019), Linggui Qihua decoction (Xiong et al., 2021), and Shunxin decoction (Jia et al., 2022) can improve myocardial hypertrophy, myocardial fibrosis, and other myocardial problems. Qishen Yiqi decoction (Huang et al., 2021) and Shengmai decoction (Zhai, 2020) can improve plasma volume expansion by reducing obesity or regulating diabetes.

### 5.2 Cellular and molecular mechanism

#### 5.2.1 Systemic microvascular inflammation

The comorbidity of HFpEF leads to systemic microvascular inflammation, including circulatory inflammation, myocardial inflammation, extracardiac organ inflammation (e.g., lung, kidney, and skeletal muscle inflammation), and diseases associated with inflammation (such as obesity and hyperlipidaemia).

The problems caused by systemic microvascular inflammation include endothelial dysfunction, oxidative stress, and decreased bioavailability. Experimental research shows that the TCM YangxinNo.2 Granules (Tang, 2016), Shenfu decoction (Huang, 2019), Xiaoqinglong decoction (Zhou et al., 2019; Zhou, 2020), Qishen Yiqi decoction (Huang et al., 2021) and Linggui Qihua decoction (Xiong et al., 2021; Xiong, 2022) can reduce pro-inflammatory cytokines TNF-α, IL-6, IL-1β, MCP-1, soluble ST2, CRP, and hs-CRP. YangxinNo.2 granules (Tang, 2016) can also increase the expression of the anti-inflammatory factor IL-10. Xindening oral liquid (Wei et al., 2017), Xiaoqinglong decoction (Zhou et al., 2019; Zhou, 2020), Shenfu Xiongze decoction (Zhang, 2020), Simiao Yongan decoction (Li et al., 2020), Qishen Yiqi decoction (Huang et al., 2021), Linggui Qihua decoction (Xiong, 2022) and Kangdaxin oral liquid (Wu et al., 2022) can reduce collagen I, collagen III, vWF, CD31, ICAM-1, VCAM-1, TGF-β1, and CD45 to improve myocardial inflammation. In addition, Xiaoqinglong decoction (Zhou et al., 2019) and Sini decoction (Zhao et al., 2022) can also regulate intestinal flora, reduce serum endotoxin, and increase acetate, propionate, and butyrate to improve extracardiac inflammation.

Systemic microvascular inflammation can activate ROS and reduce the utilization of NO and cGMP, which leads to an increase in cAMP or PKA, and an imbalance in intracellular Ca^2+^. Low availability of cGMP leads to low expression of PKG, inhibition of titin phosphorylation, and decreased myocardial compliance. On the one hand, TCM improves oxidative stress: Nuanxin capsule (Lou, 2021) and Qishen Yiqi decoction (Huang et al., 2021) can reduce the activities of ROS and MDA. Xinjierkang oral liquid (Yu et al., 2013) and Shenqi Lixin decoction (Zhuang et al., 2021) can increase the availability of NO and cGMP, improve the PKG channel, and enhance the protein and gene expression of SOD and Bcl-2. Poge Heart-Saving decoction (Liu et al., 2020) inhibits the RASS system to reduce the production of AngII. On the other hand, to improve endothelial dysfunction, Fuzi Banxia decoction (Sun et al., 2019), Shenfu Xiongze decoction (Zhang, 2020), and Xindening oral liquid (Wei et al., 2017) can inhibit important molecules P38 and MAPKAPK in the VEGF pathway. Xinjierkang oral liquid (Yu et al., 2013), Tongmai Yangxin pill (Chen et al., 2021), and Shunxin decoction (Jia et al., 2022) can enhance myocardial compliance by increasing NPRA, GC, and eNOS in the cGMP/PKG signalling pathway to increase cGMP and PKG in cardiomyocytes. Shensong Yangxin decoction (Zhang et al., 2018) can decrease PKA, RyR2, NOX2, and NOX3 to maintain Ca2+ homeostasis.

#### 5.2.2 Cardiometabolic abnormalities

Abnormal Cardiac metabolism is mainly due to myocardial energy damage, which is caused by three mechanisms: 1) abnormal structure and function of mitochondria, 2) changes in substrate utilization, and 3) intracellular calcium overload. The structural and functional abnormalities of mitochondria are reflected in organelle dyspnoea, shrinkage, enlargement, a decrease in membrane potential, and other problems, resulting in a decrease in ATP synthesis. Due to the change in mitochondrial function and structure, the utilization of substrate changes accordingly, the heart changes from fatty acid metabolism to glucose metabolism, glucose oxidation decreases, glucose degradation occurs, and lactic acid increases. At the same time, SERCA2a deficiency eventually leads to intracellular calcium overload.

Experimental studies have shown that the TCM Nuanxin capsule (Lou, 2021), Shenfu decoction (Huang, 2019), Simiao Yongan decoction (Li et al., 2020), Shunxin decoction (Jia et al., 2022), Shenqi Lixin decoction (Zhuang et al., 2021), Jiawei Shenfu granules (Liu, 2013), and Yiqihuoxue granules (Ning, 2019) can regulate the volume of mitochondria and enhance the gene and protein expression of PGC-1α, PGC-1β, ERR-α and ERR-β to protect the ultrastructure. They can also activate the AMPK pathway to inhibit phosphorylation, inhibit caspase3 and increase Bcl-2 to prevent mitochondrial apoptosis and enhance OCR and ATP to improve respiratory function. Shenfu decoction (Huang, 2019) and Gegen Qinlian decoction (Han et al., 2017) optimize the metabolism and utilization of fatty acids, glucose, and pyruvate to different degrees and enhance the expression of carnitine palmitoyltransferase 1A (CPT-1A), pyruvate dehydrogenase kinase-4 (PDK4), fatty acid translocase (CD36) and glucose transporter (GLUT4). Simiao Yongan decoction (Li et al., 2020) increases PGam1, pyruvate to reduce sugar degradation, PPAR-α, and ceramide. Linggui Qihua decoction (Xiong et al., 2021) can reduce FINS, GSP, and GLU to regulate glucose and lipid metabolism. Shensong Yangxin decoction (Zhang et al., 2018), Shengmai decoction (Zhai, 2020), Jiawei Shenfu granules (Liu, 2013), Yiqi Huoxue decoction (Chen, 2014; Liu, 2017), and Songling Xuemaikang capsule (Qi et al., 2020) can increase the expression of SERCA2a and NCX1 to reduce intracellular Ca^2+^ overload. In addition, they can inhibit RyR2 and CaMKII to reduce the release of Ca^2+^, and inhibit ERK1/2, leading to downregulation of GATA4 and improvement of BNP.

#### 5.2.3 Cellular/extracellular structural abnormalities

PKG, cGMP, and Titin phosphorylation in cardiomyocytes jointly regulate myocardial contractile motion, and the high expression of Titin in cardiomyocytes reflects the decrease in Titin phosphorylation. TCM Fuzibanxia decoction (Sun et al., 2019), Tongmai Yangxin pill (Chen et al., 2021), Qishen Yiqi decoction (Huang et al., 2021), Yiqi Huoxue decoction (Liu, 2017), and Shunxin decoction (Jia et al., 2022) can reduce PDE5A or PKA to increase eNOS and cGMP, activate the PKG pathway, regulate Titin phosphorylation to inhibit Titin, and improve myocardial compliance.

Structural abnormalities of myocardial extracellular matrix (ECM), including fibrosis and amyloid protein, can occur. ECM mainly depends on the balance of MMPs and TIMPs. If the balance is lost, myocardial fibrosis will occur. In addition, TGF-β/Smads, p38MAPK, the G protein-coupled receptor kinase (GRK) signal pathway, and TGF-β1 expression induced by AngII can all lead to the development of fibrosis. TCM Shenfu decoction (Huang, 2019), Linggui Qihua decoction (Xiong, 2022), and Shengmai decoction (Zhao et al., 2016) can regulate ECM deposition and balance the relationship between MMP-9, MMP2, TIMP-1, and TIMP-2. Xiaoqinglong decoction (Zhou et al., 2019) and Yiqi Xiefei decoction (Wang, 2019) can reduce the area of fibrosis. Shenfu Xiongze decoction (Zhang, 2020), Simiao Yongan decoction (Li et al., 2020), Linggui Qihua decoction (Xiong, 2022), Xindening oral liquid (Wei et al., 2017), Songling Xuemaikang capsule (Qi et al., 2020), and Kangdaxin oral liquid (Wu et al., 2022) can inhibit P38, MAPK, Smad3, TGF-β1, and AngII.

## 6 Conclusion and perspectives

The number of deaths caused by HFpEF worldwide is gradually increasing every year, and increasing attention has been given to the prevention and treatment of HFpEF. Western medicine treatment can control the development of HFpEF, but it still faces the bottleneck problem. While developing new drugs and devices, modern medicine has recognized the individual treatment advantages and overall regulatory effects of TCM.

At present, the integration of traditional Chinese and Western medicine has become an objective and generally accepted medical model in the treatment of HFpEF. TCM treatment can improve the clinical symptoms of HFpEF, enhance the quality of life of patients, lower the poor prognosis of patients, reduce mortality and rehospitalization rates, and provide new ideas and methods for the treatment strategy of HFpEF. However, due to the diversity of TCM syndrome types and the strong subjectivity of dialectical analysis, TCM lacks certain convincing power in the process of development. The relationship between TCM and botanical drugs lacks a rigorous scientific research system, and the relationship between the ‘monarch’ and ‘minister’ in the compound prescription of TCM is seldom discussed. In addition, most of the studies on the treatment of HFpEF with TCM have a small sample size, short observation time, and lack of rigor in design. Therefore, the research results cannot be widely applied and popularized. In the future, research on TCM in the treatment of HFpEF should start from the following three angles 1) In terms of theory, it is convenient to distinguish syndrome types by setting an objective threshold for dialectical analysis. The purpose of the research is to analyse the relationship between the existing compound prescriptions of TCM and botanical drugs. 2) From a clinical perspective, we need to standardize the clinical design; perform large-sample, multicentre randomized clinical controlled trials; enhance the observation and follow-up time; and evaluate the effectiveness and safety of TCM on HFpEF. 3) In terms of experiments, people should identify the subtypes of HFpEF and expand different modelling methods to consider different mechanisms of TCM treatment to provide new ideas for improving HFpEF.

In summary, HFpEF is still a thorny clinical disease. TCM has great potential for HFpEF. However, more extensive and rigorous clinical and animal experiments are needed to promote the application of TCM in HFpEF.

## Figures and Tables

**Table:** 

TCM	traditional Chinese medicine
HFpEF	Heart failure with preserved ejection fraction
TCMSS	TCM syndrome scale
TCMFDIS	TCM Four-Dimensional Diagnostic Information Scale
NYHA	Classification of New York Heart Association heart function
MLHFQ	Minnesota Living with Heart Failure Questionnaire
EQ-5D	EuroQol Five Dimensions Questionnaire
SAS	Self-Rating Anxiety Scale
SDS	Self-rating depression scale
FS-14	Fatigue Scale-14
6MWT	6 Minute Walk Test
CPET	Cardiopulmonary Exercise Test
UCG	UltrasoundCardiogram
STI	Speckle tracking imaging
FINS	Fasting serum lisulin
HOMA-IR	Homeostasis model assessment-insulin resistance
GLC	Glucose
GLUT	glucose transporters
GCGR	Glucagon receptor
TGF-β1	transforming growth factor-β1
PGC-1α	Peroxisome proliferator-activated receptor gamma coactivator 1-alpha
|GLS|	Global longitudinal strain
UT	Ultrasonic Test
CI	cardiac index
SI	Stroke volume index
ST2	growth stimulation expressed gene 2
LPS	Lipopolysaccharide
ALD	aldosterone
CRP	C-reactive protein
ET	endothelin
TNF-α	Tumor necrosis factor-α
Gs-α	Gs alpha subunit
MCP-1	Monocyte chemoattractant protein-1
AngII	Angiotensin II
vWF	von Willebrand Factor
PECAM-1	Platelet endothelial cell adhesion molecule
ICAM-1	intercellular cell adhesion molecule-1
VCAM-1	vascular cell adhesion molecule-1
CD36	cluster of differentiation 36
CD45	leukocyte common antigen
CPT-1A	carnitine palmitoyltransferase 1A
PDK4	pyruvate dehydrogenase kinase 4 Gene
TC	Total cholesterol
LDL-C	Low-DensityLipoproteinCholesterol
NEFA	nonestesterified fatty acid
SBP	Systolic blood pressure
DBP	Diastolic blood pressure
Hcy	Homocysteine
LW	Lung weight
HW	Heart weight
BW	Body weight
TL	tibial length
FIB	Fibrinogen
LVAWd+LVPWd	Left ventricular end-diastolic anterior wall thickness + left ventricular end-diastolic posterior wall thickness
LVEF	Left Ventricular Ejection Fraction
LVFS	Left Ventricular Fraction Shortening
LAVI	Left atrial volume index
LVMI	Left Ventricular Mass Index
IVRT	Isovolumic relaxation time
LVWT	left ventricular wall thickness
LVEDP	Left Ventricular End-Diastolic Pressure
LA/Ao	left atrial to aortic root ratio
±dp/dtmax	maximal left ventricular pressure rate of change
CaMKII	calmodulin-dependent protein kinase II
PKA	Protein kinase A
PKG	protein kinase G
cGMP	cyclic guanosine monophosphate
cAMP	Cyclic adenosine monophosphate
RyR2	Ryanodine2
NF-κB	nuclear factor kappa-B
Bax	Assaciated X protein
Bcl-2	B-cell lymphoma-2
MMPs	Matrix metalloproteinases
TIMPs	tissue inhibitor of matrix metalloproteinases
SOD	SuperoxideDismutase
MDA	Malondialdehyde
LDH	lactatedehydrogenase
cTnI	cardiac troponinI
CK-MB	Creatine Kinase-MB Form
Smad	Drosophila mothers against decapentaplegic
MAPKs	Mitogen-activated protein kinases
OTUS	Operational Taxonomic Units
ROS	reactive oxygen species
AHA	American Heart Association
ACC	American College of Cardiology
HFSA	Heart Failure Society of America
ESC	European Society of Cardiology
ACCF	American College of Cardiology Foundation.
